# Undifferentiated Febrile Illness in Kathmandu, Nepal

**DOI:** 10.4269/ajtmh.14-0709

**Published:** 2015-04-01

**Authors:** Corinne N. Thompson, Stuart D. Blacksell, Daniel H. Paris, Amit Arjyal, Abhilasha Karkey, Sabina Dongol, Abhishek Giri, Christiane Dolecek, Nick Day, Stephen Baker, Guy Thwaites, Jeremy Farrar, Buddha Basnyat

**Affiliations:** Centre for Tropical Medicine, Nuffield Department of Clinical Medicine, Oxford University, Oxford, United Kingdom; Hospital for Tropical Diseases, Wellcome Trust Major Overseas Programme, Oxford University Clinical Research Unit, Ho Chi Minh City, Vietnam; The London School of Hygiene and Tropical Medicine, London, United Kingdom; Wellcome Trust, London, United Kingdom; Mahidol Oxford Tropical Medicine Research Unit, Faculty of Tropical Medicine, Mahidol University, Bangkok, Thailand; Oxford University Clinical Research Unit–Nepal, Patan Academy of Health Sciences, Lalitpur, Nepal

## Abstract

Undifferentiated febrile illnesses (UFIs) are common in low- and middle-income countries. We prospectively investigated the causes of UFIs in 627 patients presenting to a tertiary referral hospital in Kathmandu, Nepal. Patients with microbiologically confirmed enteric fever (218 of 627; 34.8%) randomized to gatifloxacin or ofloxacin treatment were previously reported. We randomly selected 125 of 627 (20%) of these UFI patients, consisting of 96 of 409 (23%) cases with sterile blood cultures and 29 of 218 (13%) cases with enteric fever, for additional diagnostic investigations. We found serological evidence of acute murine typhus in 21 of 125 (17%) patients, with 12 of 21 (57%) patients polymerase chain reaction (PCR)-positive for *Rickettsia typhi*. Three UFI cases were quantitative PCR-positive for *Rickettsia* spp., two UFI cases were seropositive for Hantavirus, and one UFI case was seropositive for Q fever. Fever clearance time (FCT) for rickettsial infection was 44.5 hours (interquartile range = 26–66 hours), and there was no difference in FCT between ofloxacin or gatifloxacin. Murine typhus represents an important cause of predominantly urban UFIs in Nepal, and fluoroquinolones seem to be an effective empirical treatment.

Undifferentiated febrile illnesses (UFIs) are a common clinical problem in south Asia.^1^,^2^ Defined as a fever without a focus of infection on initial physical examination or in basic laboratory tests, UFIs represent a considerable burden of disease with diagnostic and therapeutic challenges. Empirical broad-spectrum antimicrobials are generally prescribed but with little evidence-based guidance on likely etiologies or potential treatment responses. Previous studies on UFIs, including those originating from our research group in Nepal,^3^–^5^ have been limited by a lack of molecular testing, little convalescent serological testing, and few data on treatment outcomes.

We sought to address this knowledge gap by expanding investigations to determine further causes and treatment outcomes of Nepalese patients with UFIs. Diagnostic tests were performed for scrub typhus, murine typhus, Spotted Fever Group (SFG) rickettsiosis, Q fever, leptospirosis, Hantavirus, *Brucella*, and dengue (Table 1). The patients described in this report were previously enrolled into a randomized, controlled trial (RCT) comparing gatifloxacin with ofloxacin for treating enteric fever in Patan Hospital, a 450-bed teaching hospital within the Kathmandu Valley, Nepal. All patients had UFIs at enrollment and came from predominantly urban regions; the methods and results from patients with subsequent blood culture-confirmed enteric fever have been reported previously.^3^ Briefly, patients were eligible to enter if they were > 2 years old, had an untreated UFI for > 3 days, and could be treated in the community. Each patient was randomly assigned to 7 days of treatment with either gatifloxacin (10 mg/kg per day in a single oral dose) or ofloxacin (20 mg/kg per day in two divided oral doses). All patients were managed as outpatients, with assessment of fever clearance time (FCT) and collection of acute (day 1) and convalescent (day 30) blood samples by trained community medical auxiliaries. Approval for the study was obtained from the Nepal Health Research Council and the Oxford Tropical Research Ethics Committee. The trial was registered as ISRCTN 63006568. Written informed consent was obtained from all study participants.

Between July of 2008 and August of 2011, 627 patients with UFIs were enrolled in the RCT: 311 of 627 (49.6%) patients received gatifloxacin, and 316 of 627 (50.4%) patients received ofloxacin (Figure 1). *Salmonella* Typhi and *Salmonella* Paratyphi A were cultured from the blood of 109 of 311 (35%) and 109 of 316 (34%) patients in each treatment arm, respectively. The remaining 409 of 627 (65%) patients had UFIs with negative blood cultures. Although no formal sample size calculation was carried out for this study, we randomly selected 125 of 627 (20%) UFI patients for additional diagnostic testing, consisting of 96 of 409 (23%) UFI patients and 29 of 218 (13%) enteric fever patients (*Salmonella* Typhi, *N* = 17; *Salmonella* Paratyphi A, *N* = 12) (Table 1).

**Figure 1.**
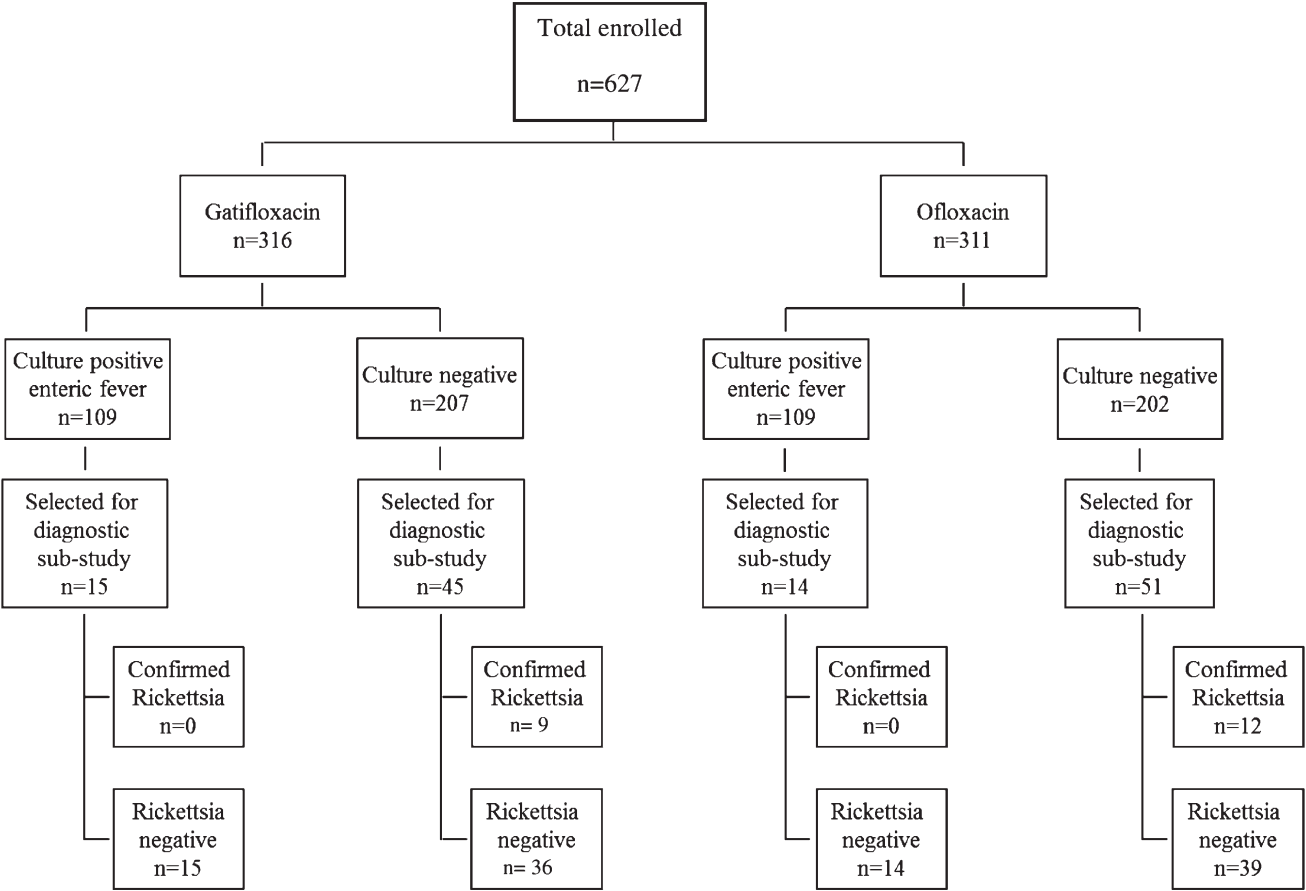
Flowchart of patients from the RCT and the substudy of UFIs. In total, 627 patients were enrolled into the clinical trial comparing gatifloxacin with ofloxacin in the treatment of enteric fever. In total, 316 patients were randomized to receive gatifloxacin, and 311 patients were randomized to receive ofloxacin. One patient was not randomized. There were 109 culture-confirmed enteric fever cases in each arm, leaving 207 and 202 culture-negative patients in the gatifloxacin and ofloxacin arms, respectively. In total, 125 patients were selected for the UFI diagnostic substudy; 29 of these 125 patients were selected from culture-positive enteric fever group, and an additional 96 patients from the culture-negative groups.

All data were analyzed using Stata v13 (College Station, TX). Kruskal–Wallis tests were used to compare clinical parameters between the enteric fever and rickettsial groups. FCTs were summarized by Kaplan–Meier estimates and compared between groups using a Cox regression model with only one covariate. All tests were performed using two-sided 5% significance.

In total, 21 of 125 (17%) patients were identified with acute murine typhus infection on the basis of at least a fourfold antibody titer rise from day 1 to day 30 (Figure 1); 10 of these cases were confirmed by quantitative polymerase chain reaction (PCR; *ompB* gene target), and 2 cases were confirmed by conventional PCR/sequencing of the 17-kDa and/or *gltA* genes. In total, 12 of 21 (57%) PCR-positive murine typhus cases were confirmed. Three cases with a *Rickettsia* spp.-positive quantitative PCR result could not be further differentiated because of limited sample specimen. However, these specimens have a high probability of being murine typhus cases because of their positive *R. typhi* serology. The possibility remains that SFG *Rickettsia* could be responsible for these cases. None of the patients with rickettsial infections were coinfected with *Salmonella* Typhi or *Salmonella* Paratyphi A. Additionally, two cases were serologically positive for Hantavirus, and one case was serologically positive for Q fever.

Although the study design allowed for limited comparison, the clinical presentations and basic laboratory values, such as complete blood count, liver function test, and creatinine, of 21 rickettsial patients and 29 enteric fever patients were, in general, similar. However, the FCT was significantly prolonged in the enteric fever patients, with a median of 88 hours (interquartile range [IQR] = 54–116), for both drugs compared with the FCT in those with rickettsial infections, with a median of 44.5 hours (IQR = 26–66; hazard ratio = 3.71; *P* < 0.001).

Our study has a number of limitations. First, we were unable to test the whole study population for alternative causes of UFI, and the 20% proportion of patients selected may not have been truly representative of the whole population. Second, serological testing for *Rickettsia* may lack specificity, although we defined acute infection as a greater than or equal to a fourfold rise in reciprocal antibody titers between admission and convalescence sera.

Despite these limitations, our study highlights that *Rickettsia* spp. are an important cause of UFIs in Nepal^6^ and that these patients present with similar clinical characteristics to enteric fever. Although the original study was designed to enroll typhoid patients and represents more of an urban population, we detected a 17% murine typhus case rate and a possible 2% *Rickettsia* spp. infection rate in a random subselection of the study. Notably, we have evidence suggesting that Hantavirus and Q fever contribute to UFIs. The absence of scrub typhus is likely because of the predominantly urban patients enrolled in this study.

The recommended therapy for murine typhus is doxycycline,^7^ although fluoroquinolones are known to be an effective alternative for the treatment of SFG rickettsioses.^8^ Without control groups of untreated or doxycycline-treated patients, only tentative conclusions can be drawn, but despite previous reports of poor responses to ciprofloxacin in murine typhus^9^,^10^ our findings suggest that gatifloxacin and ofloxacin may be effective empirical treatment choices in Nepalese patients with UFIs.

## Figures and Tables

**Table 1:** Diagnostic tests used for the study

Organism/diagnostic tests	Supplier	Catalog number	Diagnostic criteria	Methodological reference or validation study	Purpose
*Orientia tsutsugamushi*					
IgM ELISA	NMRC	In house	≥ 0.2 nett OD	11	Serological screening
IgG ELISA	NMRC	In house	≥ 0.2 nett OD	11	Serological screening
IgM IFA	ARRL	RT-001	≥ Fourfold rising titer in paired samples	12	Quantitative serological confirmation
IgG IFA	ARRL	RT-001	≥ Fourfold rising titer in paired samples	12	Quantitative serological confirmation
Real-time PCR	MORU	In house	47-kDa gene amplification	13	
*R. typhi*					
IgM ELISA	NMRC	In house	≥ 0.2 nett OD	11	Serological screening
IgG ELISA	NMRC	In house	≥ 0.2 nett OD	11	Serological screening
IgM IFA	ARRL	RT-001	≥ Fourfold rising titer in paired samples	12	Quantitative serological confirmation
IgG IFA	ARRL	RT-001	≥ Fourfold rising titer in paired samples	12	Quantitative serological confirmation
Real-time PCR	MORU	In house	ompB gene amplification	14	Confirmation of infection
*Rickettsia* spp.					
Real-time PCR	MORU	In house	17-kDa gene amplification	13	Confirmation of infection
*Coxiella burnetti*					
Phase II IgM ELISA	Serion	ESR1312M	Manufacturer's criteria	Product insert	Serological screening
Phase I/II IFA	Fuller		Manufacturer's criteria	Product insert	Quantitative serological confirmation
Hantavirus Puumala					
IgM ELISA	Serion	ESR145M	Manufacturer's criteria	Product insert	Serological screening
Anti-Hantavirus IIFT Mosaic II Test	Euroimmun				Quantitative serological confirmation
*Leptospira*					
IgM ELISA	Serion	ESR125M	Manufacturer's criteria	Product insert	Serological screening
Microscopic agglutination test*	QSHL	In house	≥ Fourfold rising titer in paired samples	15	Quantitative serological confirmation
*Brucella* spp.					
Rose–Bengal	NIAH	In house	Positive agglutination reaction	16	Serological screening
Dengue					
SD NS1 Ag ELISA	Alere	11EK50	Manufacturer's criteria	17	Serological screening

ARRL = Australian Rickettsial Reference Laboratory; ELISA = enzyme-linked immunosorbent assay; IFA = indirect immunofluorescence assay; Ig = immunoglobulin; IIFT = indirect immunofluorescence test; MORU = Mahidol Oxford Research Unit; nett OD = net optical density (net stands for the difference from baseline to measured values); NIAH = National Institute of Animal Health–Thailand; NMRC = Naval Medical Research Centre; QSHL = Queensland State Health Laboratory; SD NS1 Ag = standard diagnostics non-structural protein number one (refers to Dengue virus protein) antigen.

* Leptospira serovars tested: pomona, hardjo, tarassovi, grippotyphosa, celledoni, copenhageni, australis, pyrogenes, canicola, hebdomadis, sari, sarmin, autumnalis, cynopteri, ballum, bataviae, djasiman, javanica, panama, shermani, and pohnpei.
